# Methods of Disease Risk Analysis in Wildlife Translocations for Conservation Purposes

**DOI:** 10.1007/s10393-016-1134-8

**Published:** 2016-06-10

**Authors:** Matt Hartley, Anthony Sainsbury

**Affiliations:** 1 University of Chester, Parkgate Road, Chester, CH1 4BJ UK; 2 Institute of Zoology, Zoological Society of London, London, UK

**Keywords:** disease, risk, analysis, assessment, translocation, conservation

## Introduction

Risk analysis processes have been developed to provide an objective, repeatable, transparent and documented assessment of the risks posed by a course of action or chain of decisions. Standardised techniques have been developed and are utilised routinely to aid decision making by governments and international organisations such as the OIE (World Organisation for Animal Health) in assessing the risk from disease to humans, domestic animals and wildlife.

Wildlife managers and decision makers are increasingly adopting these processes to aide management of disease threats to conservation interventions, such as reintroductions, rehabilitation and release or wild-to-wild translocations. The ability to use structured, reasoned, recognised qualitative approaches is particularly useful when evidence and data are lacking, which is common when working with wildlife. Several different systems and formats are in use, but all broadly follow the principles of risk analysis advocated by Covello and Merkhofer (1993) in their treatise on across-discipline risk analysis for the above benefits to be realised.

This paper reviews approaches to disease risk analysis in wildlife translocation projects addressing reasons for undertaking assessments, potential sources of information and personnel involved. There are always multiple hazards, which complicates the traditional risk analysis approach, and paucity of information on the identity and geographical distribution of parasites hampers hazard identification (Sainsbury and Vaughan-Higgins 2012).

The Zoological Society of London’s Disease Risk Analysis and Health Surveillance (DRAHS) project has been operating for 25 years, in partnership with Natural England and non-governmental organisations, to assess and respond to disease risks associated with interventions undertaken for the national Species Recovery Programme for native wildlife. Our experience from conducting these disease risk analyses is used to describe the limitations of the analysis and propose some methods to respond to these difficulties.

## Disease Impacts of Wildlife Translocations

Translocation is the intentional movement of living organisms from one geographical area for free release into another with the object of establishing, re-establishing or augmenting a population (Kock et al. 2010).

The Natural England Species Recovery Programme has utilised a range of different translocation methods over the last 25 years. These include (i) captive breeding and release to supplement diminished populations, for example, in hazel dormice (*Muscardinus avellanarius*) and corncrakes (*Crex crex*), (ii) Importation of animals from other countries to re-establish populations in the UK such as for the pool frog (*Pelophylax lessonae*) and short-haired bumble bee (*Bombus subterraneus*) or (iii) wild-to-wild translocations to establish new local populations and increase a species’ range such as for wart-biter cricket (*Decticus verrucivorus*) and smooth snake (*Coronella austriaca*).

In the past, wildlife translocations were commonly undertaken without thought to disease issues (Griffin et al. 1993). Indeed, the DRAHS project was established in 1989 many years after the first translocations had been undertaken for the Species Recovery Programme.

The potential impact of infectious disease on the outcome of wildlife conservation interventions has only recently been recognised. Disease may be seen in the focus species or in other wild or domestic species or humans at the site of the intervention or may have wider environmental or ecosystem effects. The impacts of a disease outbreak may affect a wide range of stakeholders such as government, farmers, local residents and businesses. For example, an unauthorised introduction of European beavers posed the potential risk of introducing the zoonotic pathogen *Echinococcus multilocularis* to the UK (Simpson and Hartley 2011).

Where the translocated species originates from an ex situ population, there is a risk that it acquires, and becomes a symptomless carrier of, infectious agents novel to the destination. Animals in ex situ environments may be mixed with species from unrelated geographic origins and as a result may be exposed to exotic (alien) pathogens and to infectious agents transmitted by carers and other humans. Furthermore, captivity, or management of ex situ populations, subjects species to stress resulting in immunosuppression and increased susceptibility to disease (Kock et al. 2010). For example, hazel dormice were exposed to a suspected novel cestode species in captivity prior to reintroduction in England (Peniche et al. 2016).

Translocated animals may lack acquired immunity or resistance to the infectious agents which will challenge them at the release site. Many diseases and parasites are highly localised in distribution as a result of the specific ecological requirements of the pathogen and/or vectors (Kock et al. 2010). For example red squirrels (*Sciurus vulgaris*) reintroduced in England were exposed to squirrelpox virus (harboured by the alien invasive grey squirrel, *Sciurus carolinensis*) at the destination reintroduction site, resulting in a severe squirrelpox disease outbreak (Carroll et al. 2009).

These examples highlight the burden of responsibility that managers of conservation interventions have when planning a project and the importance of a robust, transparent and comprehensive process to identify potential disease risks and to manage those risks appropriately and effectively.

## What is Disease Risk Analysis?

Risk analysis is a tool intended to provide decision makers with an objective, repeatable and documented assessment of the risks posed by a particular course of action (MacDiarmid, 1997). As the approach has developed and diversified, a more specific disease focused definition was proposed by Jakob-Hoff et al. (2014) who stated that disease risk analysis is a structured, evidence-based process that can help in decision making in the face of uncertainty and determine the potential impact of infectious and non-infectious diseases on ecosystems, wildlife, domestic animals and people. The authors explained how the results from disease risk analysis can be used to help decision makers to consider an evidence-based range of options for the prevention and mitigation of disease in the population(s) under consideration.

## Development of Disease Risk Analysis

**Figure 1:**
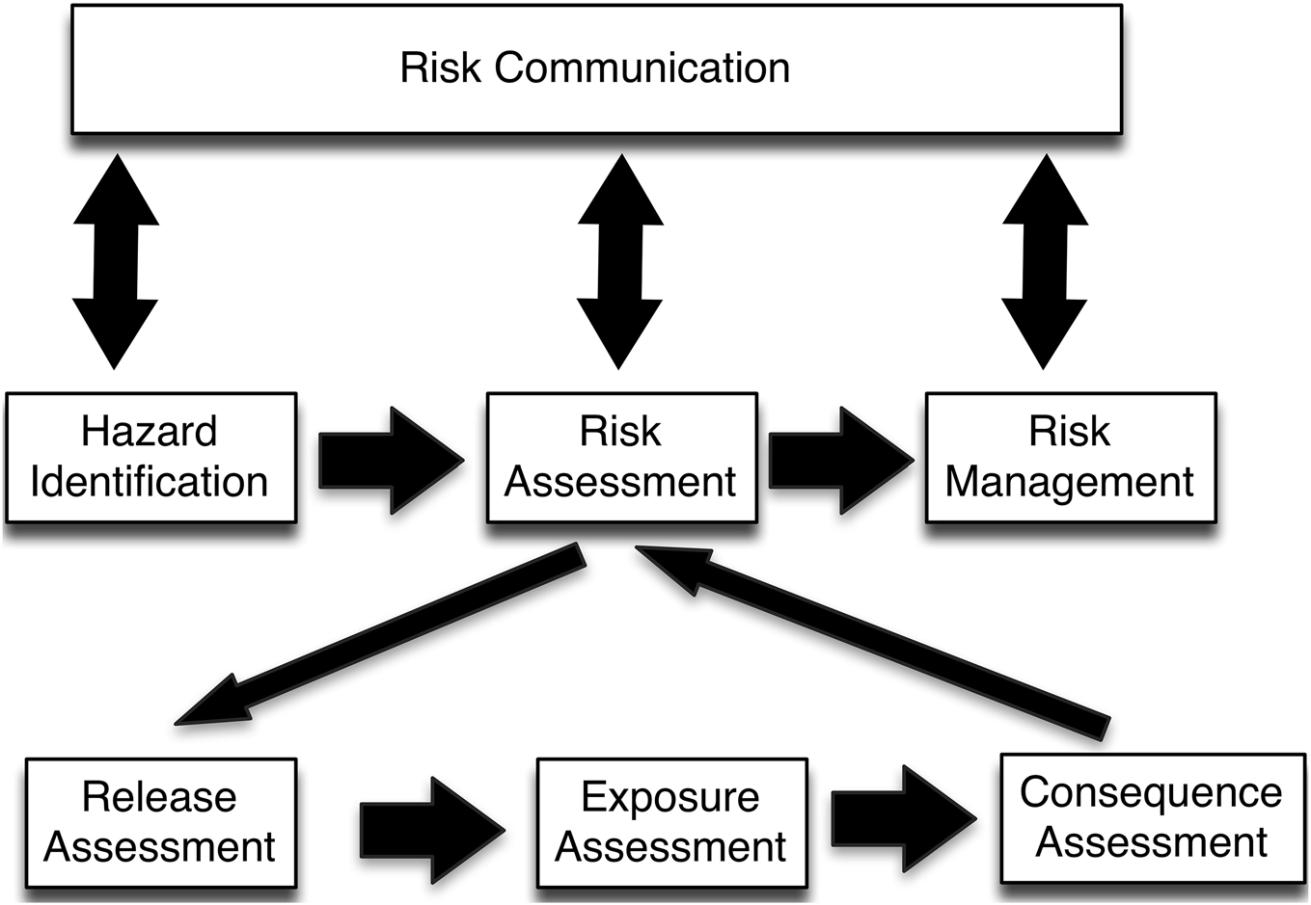
Risk analysis framework.

Disease risk analysis was developed by adapting environmental risk analysis techniques. The World Organisation for Animal Health (OIE) developed the OIE Risk Analysis Framework (Murray 2004). This has formed the basis of disease risk analysis systems developed by other organisation such as the Department of Environment, Food and Rural Affairs and Biosecurity Authority, Ministry of Agriculture, New Zealand. The framework has been used for a range of scenarios beyond import risk analysis including domestic animal notifiable disease incursion, wildlife disease control (Hartley 2009; Hartley et al. 2012) and pest species entry (Tana and Daldry 2003). An example of a risk analysis system, including the risk assessment process, is shown in Fig. 1.

Davidson and Nettles (1992), Leighton (2002), Armstrong et al. (2003) and Miller (2007) devised qualitative methods for assessing the risks of disease associated with wildlife translocations. Armstrong et al. (2003) and Miller (2007) also devised quantitative methods. In 2014, the IUCN and OIE jointly published the Manual of Procedures for Wildlife Disease Risk Analysis, which collated and consolidated current knowledge and provided a framework for developing, interpreting and utilising disease risk analysis in wildlife conservation.

## Disease Risk Analysis Approaches and Modifications for Wildlife Translocation

The first stage in any risk analysis is to determine the problem or issue, which is to be addressed, otherwise known as the ‘risk question’. The risk question needs to clearly establish the goals, scope and focus of the analysis and will depend on who has commissioned the work and is the risk manager. The results of disease risk analysis for wildlife translocations may be used by the conservation team running the project, to identify risks to the threatened species of focus and increase the likelihood of project success. A risk analysis commissioned by a governmental agency authorising and licencing a wildlife translocation may prioritise potential risks to other wildlife, including at the destination site, whereas public health officials will primarily be interested in zoonotic risks associated with the translocation. Government agricultural agencies, farmers and landowners will have a focus on potential risks to domestic animals and agricultural production (Hartley and Gill 2010).

In practice, a single disease risk analysis is likely to be required to meet the requirements of all of these stakeholders. The DRAHS project risk analysis, when commissioned at the initiation of a project, will not only guide resources and activities of the Natural England Species Recovery Programme but contribute to official licensing decisions and cross-governmental support.

Even when focused solely on threatened species, there are a wide range of scenarios in which disease risk analysis could be used including (i) prior to commencing a reintroduction programme (Sainsbury et al. 2012), (ii) in response to a specific disease identified during the course of a project or (iii) in response to an epidemiological investigation of unknown disease in the focus species. Table 1 describes how DRA, disease risk management (DRM) and post-release health surveillance (PRHS) have been built into the health and disease monitoring of species translocations covered by the DRAHS project.

**Table 1:** Overview of Disease Risk Analysis (DRA), Disease Risk Management (DRM) and Post-release Health Surveillance (PRHS) Conducted by the Disease Risk Analysis and Health Surveillance (DRAHS) Project for Species Subject to Translocation in England.

Species	Translocation description	Scenario disease risk analysis (DRA) undertaken	Disease risk management (DRM) actions	Review process for disease risk analysis
Corncrake *Crex crex*	Captive breeding and release from two zoological collections	DRA not completed—programme initiated before DRA was built into DRAHS project	Quarantine. Biosecurity protocol; Specific actions undertaken to combat diagnosed disease, for example coccidiosis, disease associated with *Enterococcus hirae*, metabolic bone disease. Pre-release health assessment	Annual revision of DRM protocols based on results
Hazel Dormice *Muscardinus avellanarius*	Captive breeding and release from over ten captive collections	DRA not completed because programme initiated before DRA was built into DRAHS project	Quarantine. Biosecurity protocol; Pre-release health assessment; elimination of suspected alien cestode parasite; therapeutic treatment for native parasites; and post-release health surveillance (PRHS)	Annual revision of DRM and PRHS protocols based on results
Sand Lizard *Lacerta agilis*	Captive breeding and release from multiple captive collections	DRA completed 32 years after the first reintroduction	Quarantine; biosecurity protocol; Pre-release health assessment, therapeutic treatment to reduce nematode infestation; post-release health surveillance	Annual revision of DRM and PRHS protocols based on results
Eurasion crane *Grus grus*	Import of eggs from Germany and captive rearing and release	DRA during planning stages of project	Quarantine; biosecurity protocol; Pre-release screening for inclusion body disease virus of cranes; pre-release health assessment; disease control for example preventive coccidial treatment; post-release health surveillance	Continuous review of DRM and PRHS protocols based on results
Wart-biter Cricket *Decticus verrucivorus*	Wild-to-wild translocations	DRA during planning stages	Quarantine; Pre-release health assessment; Post- release disease surveillance	Annual review of DRM PRHS protocols based on results
Fen raft Spider *Dolomedes plantarius*	Captive breeding and release from multiple zoos	Reintroduction had started before a DRA could be completed	Quarantine; biosecurity and hygiene protocol; pre-release health assessment	Annual review of DRM protocol based on results
Smooth Snake *Coronella austriaca*	Wild-to-wild translocations	DRA completed 40 years after translocation programme started	Quarantine; biosecurity protocols; pre-release health assessment; post-release health surveillance	Annual review of DRM PRHS protocol based on results
Pool frog *Pelophylax lessonae*	Wild-to-wild translocation across geographical and ecological barriers from Sweden to England. Wild-to-wild translocation from first site in UK to second site	DRA completed before first translocation. DRA revised prior to translocation to second site	Quarantine; biosecurity protocol; pre-release health assessment; post-release health surveillance of pool frogs and sympatric amphibian species	Annual review of DRM PRHS protocols
European adder *Vipera berus*	Proposed captive breeding and reintroduction. Reintroduction did not proceed	DRA completed. Revision of reintroduction plan recommended	No reintroduction	Not applicable
Short-haired bumblebee *Bombus subterraneus*	Wild-to-wild translocation from Sweden to England across geographical and ecological barrier	DRA completed prior to translocation. DRA revised when new infectious agents detected in source population or new hazard suspected	Quarantine; biosecurity protocols; screening for alien parasites prior to release; bees with alien parasites not released (may be returned to source)	Annual review of DRM and PRHS protocols based on results
Cirl bunting *Emberiza cirlus*	Initial plan to captive breed in zoo ceased following DRA. Wild-to-wild translocation	DRA completed before translocation	Quarantine. Biosecurity protocol. Pre-release health assessment. Preventive therapeutic treatment for coccidial parasites; post-release health surveillance	Annual review of DRM PRHS protocols
Red kite *Milvus milvus*	Translocation eggs and chicks from Sweden and Spain across ecological and geographical barriers	No DRA – programme commenced prior to implementation of DRA in DRAHS project	Quarantine; biosecurity protocols; pre-release health assessment; preventive treatment for parasite infestations; post-release health surveillance	Annual review of DRM PRHS protocols
White-tailed sea eagle *Haliaeetus leucocephalus*	Proposed reintroduction from Poland. No reintroduction occurred	DRA completed prior to proposed reintroduction	Quarantine; biosecurity protocol; pre-release assessment; post-release health surveillance protocol written	Not applicable
Fishers estuarine moth *Gortyna borelii lunata*	Captive breeding in a zoological collection and reintroduction	Reintroduction commenced prior to DRAHS involvement. No DRA	Quarantine; biosecurity protocols; pre-release health assessment; post-release health surveillance	Annual review of DRM PRHS protocols
Red squirrel *Sciurus vulgaris*	Trial wild-to-wild translocation	Translocation occurred before DRAHS integrated DRA into monitoring programme	Quarantine; biosecurity protocols; pre-release health assessment; therapeutic preventive treatment for parasites; post-release health surveillance	Annual review of DRM PRHS protocol
Barbary carpet moth	Proposed captive breeding and reintroduction. Reintroduction did not occur following DRA	DRA completed prior to proposed reintroduction	Not applicable	Not applicable
Red-barbed ant *Formica rufibarbis*	Captive breeding and reintroduction from two captive collections	DRA completed prior to translocation	Quarantine; biosecurity protocols; pre-release health assessment; post-release health surveillance	Annual review of DRM PRHS protocol based on results
Field cricket *Gryllus campestris*	Captive breeding and reintroduction from one captive collection	DRA completed after reintroduction commenced	Quarantine; biosecurity protocols; pre-release suspected alien parasite screening; post-release health surveillance	Annual review of DRM PRHS protocol

Defining the risk question is sometimes included within the first step of the risk analysis framework along with hazard identification (e.g. US Environmental Protection Agency (US EPA) 1998). However, in some circumstances, the risk managers may define the problem description before commissioning the work and appointing risk assessors. Conducting separate problem description and hazard identification exercises helps protect the scientific evaluation of risk from being overly influenced by political and social issues that may arise during problem description (US Environmental Protection Agency (US EPA) 1998).

Once the risk question has been determined, hazard identification is undertaken. A hazard can be defined as a biological, chemical or physical agent in, or a condition of an animal, or an animal product with the potential to cause an adverse effect on health (Jakob-Hoff et al. 2014).

Generally in traditional domestic animal import and incursion disease risk analysis, developing the risk question will result in two different scenarios. Either the problem is focused on a single risk pathway but involves multiple hazards, for example, risks posed to consumers of legally imported cooked chicken from outside of Europe or the problem is specific to one well-defined hazard, and there are multiple risk pathways, for example, rabies entering the country in domestic dogs. However, this is very rarely the situation when considering risks posed by wildlife translocations. Risk assessors working with wildlife health problems have lead the development of techniques for working with multiple hazards and multiple risk pathways in a single risk analysis (Hartley 2009; Hartley et al. 2012; Sainsbury et al. 2012) .

Once a problem has been described, it will be possible to estimate the level of detail required in the risk analysis. Criteria could be established for ranking the importance of each hazard and its possible direct and indirect consequences within the bounds of the defined problem. This prioritisation step is important as the number of pathogens harboured by every organism could potentially make the risk analysis enormous and therefore unrealistic and unachievable with the resources available. For example, Neimanis and Leighton (2004) analysed qualitatively the risks of disease from 122 species of parasites associated with translocation of wild Eastern Turkeys *(Meleagris gallopavo)* to Canada. A qualitative analysis of risk of disease from so many parasites is difficult and time consuming, and a quantitative analysis is not feasible (Sainsbury et al. 2012). When undertaking hazard identification during preparations to translocate elk *(Cervus elaphus)*, Corn and Nettles (2001) identified 190 potential pathogens but prioritised 16 hazards considered to be of a higher risk than negligible or very low. DRAHS conducted qualitative analysis for 26 source, destination, carrier and transport hazards for the short-haired bumblebee *Bombus subterraneus* translocation (Vaughan-Higgins et al. 2012); 18 carrier, transport, source zoonotic and destination hazards for smooth snake *Coronella austriaca* translocation (and discounted a further 22 suspected hazards) (Masters and Sainsbury 2011); 16 carrier, source, destination, zoonotic and population hazards for proposed translocation of the European adder *Vipera berus* (Beckmann et al. 2014a) and 21 source and destination hazards for the proposed translocation of white-tailed sea eagle *Haliaeetus leucocephalus* from Poland to England (Sainsbury et al. 2010).

Prioritisation of hazards is extremely difficult in wildlife translocation scenarios as the epidemiology of many known pathogens is poorly understood and unknown pathogens may be present but undetected. Many catastrophic disease outbreaks as a consequence of translocation have been associated with previously unknown parasites (Bobadilla Suarez et al. 2015; Walker et al. 2008). It is difficult to predict the consequences of infection in immunologically naïve animals, and disease surveillance data are limited, so the presence or absence of a pathogen in the source population or in animals at the destination site may not be known.

In response to this, in the DRAHS project, Sainsbury et al. (2012) modified the definition of a hazard to better reflect the epidemiological scenarios of wildlife translocation. Host–parasite encounters may occur at several stages of the translocation pathway, non-infectious diseases can have negative effects on the translocated population and other stressors may trigger disease. Previous definitions of a hazard require an infectious agent to cause harm (Murray 2004). Understanding of parasite pathogenicity in wild animals is limited, but given knowledge of the threat posed by non-native invasive parasites, Sainsbury et al. (2012) considered that novelty of an infectious agent to the host is a sufficient reason to classify the infectious agent as hazardous in the absence of information on pathogenicity. These authors defined hazards on the basis of whether a parasite was new to a host, on the immunological interactions between host and parasite, the effect of stressors on these interactions or the ability of the parasite to affect populations.

**Figure 2:**
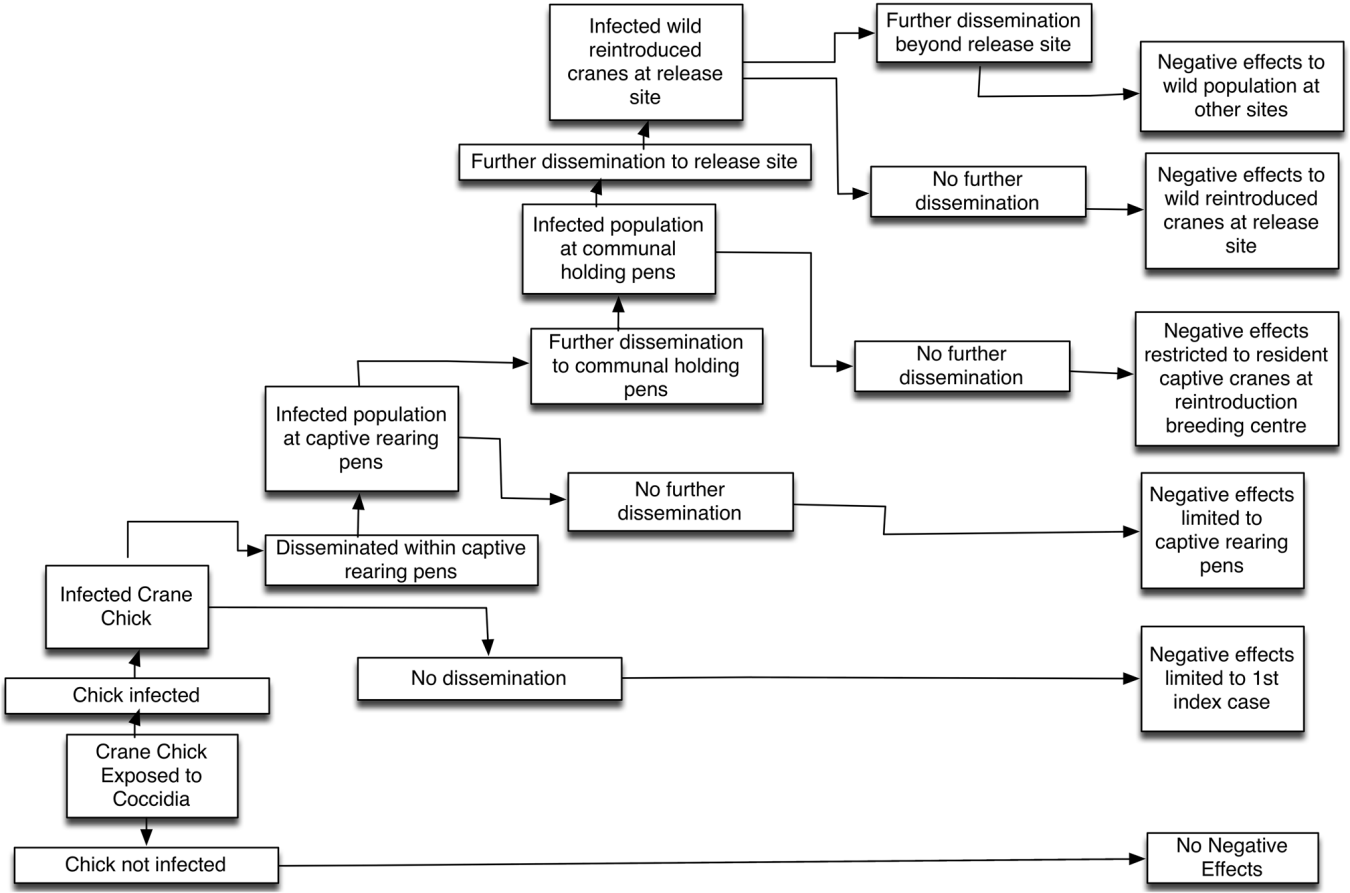
Scenario tree illustrating the effects of coccidia infection on eurasian cranes reintroduced to England from Germany. Modified from Sainsbury et al. (2012).

Once the hazard identification process has been completed the risk pathways or scenario trees can be developed. These graphical models identify the various factors involved in the risk assessment process and the various biological pathways of expected events resulting in the occurrence of a defined outcome. Thus, these visual pictures provide a useful conceptual framework for the risk assessment, facilitate transparency and aid in communicating the risks to the various stakeholders, in a simple, logical and reasoned framework (MacDiarmid and Pharo 2003). Scenario trees can be constructed for the release, exposure and consequence assessment steps in the risk assessment process: release. An example is provided in Fig. 2.

Wildlife translocation risk assessment should follow the basic scientifically accepted approaches which have been developed to ensure that the output is valid, transparent and accepted. The framework for risk assessment, being composed of release, exposure, and consequence assessments, is established and described by several authors whom have adhered to the same concepts with modification to the particular scenarios in their individual fields (Covello and Merkhofer,^1993^; Murray 2004; Jakob-Hoff et al. 2014, Sainsbury et al. 2012). A model of risk assessment is shown in Fig. 1.

Import disease risk assessment as advocated by the OIE uses international borders as the division between source and destination environments and thus limits the possibility of hazard release and exposure (Murray 2004). Wild animals and their parasites are restricted in their distribution by ecological barriers (e.g. niche separation) and topographic barriers (e.g. mountain ranges or seas) rather than political barriers, and this must be reflected in the risk assessment (Sainsbury and Vaughan-Higgins, 2012).

In order to ensure utmost transparency and to aide development of the risk assessment, a risk table is a useful tool. This is particularly true where multiple hazards and multiple risk pathways occur. A risk table shows the steps in the risk pathway, summarises evidence, states the release, exposure and consequence assessment, and produces a final risk estimation for the hazard or pathway (Hartley 2009; Hartley et al. 2012; Jakob-Hoff et al. 2014).

The risk assessment can be developed using a wide range of additional tools, ranging from simple diagrams and spreadsheets to more clearly present the data, to bespoke software which develops quantitative assessments using data collected, to complex models which explore variability and uncertainty. A comprehensive review of available risk assessment tools is presented by Jakob-Hoff and others (2014). The use of these tools does not divert from the risk analysis framework but merely contributes to enabling risk conclusions to be reached and justified. In many wild animal translocation scenarios, the lack of understanding of hazard epidemiology prevents accurate calculation of probability of disease occurrence and limits the value of the tools.

Risk assessment is an iterative process. As the assessment is built, it may be recognised that some potential hazards have been missed or one of the risk pathways is not as first thought. For example, black queen cell virus (BQCV) was detected in short-haired bumblebees in Sweden in the second year of the reintroduction programme but had not previously been considered a hazard. The DRA was immediately updated with a risk assessment of BQCV as a source hazard (Shotton and Sainsbury 2015). It is important to modify the analysis to represent the best current knowledge. This modification may occur throughout the life of a translocation project as new information from the literature or the project itself becomes available. In the DRAHS project, the risk assessments may be reviewed many times during a project and are often reviewed on an annual or bi-annual basis.

In addition, post-translocation disease surveillance, disease outbreak investigation findings and post-mortem examination results from the focus species or other animals at the translocation site or the population of origin are fed back into the risk assessment. For example, in 2013, eleven corncrakes with metabolic bone disease were identified at their pre-release health examination and changes made to their diet over the following 18 months to try to reduce the incidence (Beckmann et al. 2014b). The risk of ranaviral disease in pool frogs was re-analysed in 2015 because further data were available on the distribution of the virus in the UK (Shotton and Sainsbury 2015). This adaptive and ongoing risk analysis is essential to be able to respond to the dynamic ecology of the populations that are worked with on this project and continually improves the performance and accuracy of the risk analysis.

## Who Should Be Involved ?

In developing risk assessments, a broad range of expertise may be required such as epidemiologists, ecologists, diagnostic scientists and conservation field staff. It is unlikely that all this expertise will be incorporated in a single ‘unit’. Therefore, risk analysis should be treated as a project with the people having the necessary skills being assembled into the team as required and consulted as necessary.

It has been stated that in order to ensure the risk assessment process is not influenced by personal or public pressures, those undertaking the assessment should not be decision makers or be influenced by decision makers (NRC 1994; Leighton 2002). While this might be a long-term ideal, in reality this is not practical as many people, especially in the wildlife field, have multiple roles and responsibilities and differing influence on decision making. Indeed decision makers may contribute important evidence in relation to likelihood and feasibility of courses of action, which will influence the translocation pathway chosen. Jakob-Hoff et al. (2014) take an alternative approach and recommend that ideally a well-prepared and well-funded workshop, in which an appropriate range of experts, stakeholders and decision makers are gathered for a facilitated, structured review and analysis of the scenario, is organised.

In the DRAHS project, wildlife veterinarians, epidemiologists, diagnostic scientists, pathologists and ecologists from ZSL work together with ecologists from Natural England to produce the DRA and DRM. A bi-annual steering committee comprising decision makers, partner representatives and technical staff with ecological, veterinary and policy experience contribute to the development and review of DRAs and challenge the risk assessments. This steering committee will also engage input from other sources such as licencing and animal health officers as needed.

## Information Required

In order to undertake a comprehensive risk assessment, a wide variety of information is required. This includes data on the species and populations of animals and parasites (including pathogens) in the source and destination populations, mechanisms of spread, potential impact, non-infectious hazards, preventative health procedures such as quarantine and pathogen screening proposed by the translocation team and post-release monitoring to be undertaken. Broader information on the ecology of the species being translocated and ecosystem such as natural geographical and ecological barriers, habitat, climate, and vegetation type may be important. Gathering this information will require a thorough review of the published literature and interrogation of unpublished sources of information such as from diagnostic laboratories, experts, researchers and field reports.

As there is invariably a paucity of data in wildlife translocation risk analysis, the use of a wide range of stakeholder expert opinion is useful. However, consultation with experts should be done in a formal and structured manner such as a facilitated workshop or a questionnaire so that the information collected is equally balanced and transparently presented in the risk analysis. Expert opinion can be developed further to help develop probability data for both qualitative and quantitative risk analysis (Murray 2004).

Information for risk analysis for the DRAHS project comes from different sources depending on the nature of the project and the purpose for which the DRA is being undertaken. Sources may include peer-reviewed literature, grey literature reports from other translocation projects, expert opinion or active parasite surveillance from the project itself, for example, in conducting the DRA for the short-haired bumblebee (Vaughan-Higgins et al. 2012).

## Quantitative Versus Qualitative Analysis

In other fields, such as environmental contamination assessment, mathematical modelling is used to generate numerical estimates of probability and the extent of negative consequences. In order to address scenarios where data are not available to feed the models qualitative approaches have been introduced and have been by far the most frequently used approach for wildlife disease risk analysis.

Accurate estimates of parameters as fundamental as prevalence of infection, incubation period, duration of infection, and the size and distribution of wildlife populations rarely exist for wild animals and their parasites. This extreme rarity of numerical data means qualitative risk assessment is probably as accurate as quantitative risk assessment in wildlife translocation.

In 25 years of the DRAHS project, it has never been possible to undertake a quantitative risk assessment as reliable numerical data were not available for the 17 species for which DRA or DRM has been conducted as a component of the Species Recovery Programme.

It should be remembered that the term qualitative risk assessment does not mean that no numerical data are used to assess the risk, but that the risk estimation is presented in words that describe the evaluated risk. The advantage of the risk assessment being presented in qualitative terms is that the use of plain language and logic is more understandable by a wider range of stakeholders and decision makers.

## Uncertainty and Subjectivity

Historically, the view was taken that if there is insufficient information the risk assessment process should be halted (Leighton 2002). However, in the field of wildlife health such an approach is not realistic as invariably, there are large gaps in data, and so few assessments could be completed. Extrapolations can be made from the best available information. It is important to state clearly the areas and extent of the uncertainty. This is almost as important as giving estimates of risk, particularly in the early stages of assessment when uncertainties may be large and the data poor.

Some risk assessors include an explanation of the reasons for uncertainty at each stage in the risk pathway (Hartley 2009; Hartley et al. 2012), which aids in transparency and enables risk managers to make separate decisions on different components of the problem where the level of acceptable risks may be different.

Disease risk analysts have not, to date, used mathematical or modelling approaches to address uncertainty. One approach that could be considered is information gap theory. This was invented by Ben-Haim (2001) to assist in decision making when there are severe knowledge gaps and when probabilistic models of uncertainty are unreliable, inappropriate or unavailable. It requires three main elements: a mathematical process model, a performance requirement and a model for uncertainty. These techniques have not been used due to the lack of collaboration between disease experts and modelling experts and the fact that disease risk analysis for translocation does not have a single desired or preferred outcome, but many different possible outcomes which means that models would need to be very complex and therefore very time consuming.

The risk assessment can highlight specific data inadequacies and deficiencies and allow sensible targeting of resources to collect essential data to improve knowledge. Therefore, the lack of good data is not a good argument for not undertaking a risk assessment (Wooldridge 2000). This is certainly true in the DRAHS project where risk analyses are reviewed by the steering committee on a bi-annual basis and decisions made on resource priorities for work over the next six months. The DRA for pool frog reintroduction recommended the collection of data on ranaviral distribution and presence (Shotton and Sainsbury 2015), and data are currently being collected.

In theory, risk analysis is an objective process. The reality is that in wildlife translocation disease risk analysis, there are often so few data available that the analyst has to substitute value judgements for facts. This is supported by the common use of expert opinion.

The use of expert opinion can generate uncertainty where there is expert disagreement. In this case, it is necessary to explore the implication of the judgements to determine their impact on the final conclusions. Experts may disagree on the body of knowledge or draw different inferences from an agreed body of knowledge. In either case, this should be reflected in the risk assessment. Much has been written concerning structured methods of eliciting expert opinion for decision making processes (Clemen 2001; Meyer and Booker 1991) all of which is relevant to using experts as a source of information for disease risk analysis.

Risk assessment may be criticised because some of its inputs are based on assumptions. However, all decision making is based on assumptions, and uncertainty and subjectivity do not mean that valid conclusions cannot be drawn. Although many of the inputs of a risk assessment are surrounded by uncertainty, one may be able to have confidence that the ‘true risk’ is unlikely to exceed the estimate resulting from a careful and conservative analysis (MacDiarmid 2001).

Wildlife translocation disease risk assessments are seldom completely objective and therefore transparency, by recording and highlighting uncertainty and subjective decisions, and the basis that these decisions have been reached is essential. In this way, as more data become available or the project is actioned and outcomes are known, the risk assessment can be revisited and revised in light of the new information.

## Disease Risk Management

Disease risk management is the process of identifying measures that can be applied to the problem which reduce the level of risk from disease. In some circumstances, risk management is not the role of the risk analysts; they are merely asked to assess the risk but not revise the assessment by proposing management actions. For example, when determining import policy for rabies susceptible zoo animals, epidemiologists and zoo veterinarians developed the risk assessment, while the import policy was determined by policy officials using the risk assessment as an evidence base (Hartley and Roberts 2015). In wildlife translocation risk analysis, it is more common for the risk assessment to be used as a tool to develop risk management actions. Consideration of risk management actions will help prioritise the hazards and redefine the acceptable levels of risk. A high-risk hazard may be readily managed and thus the risk reduced allowing a decision to proceed with the intervention to be made. The description and particularly visualisation of the risk pathway greatly aids the identification of critical control points where risk management actions can be applied (Hartley and Schmidt 2013) and was used in the DRA for the reintroduction of the cirl bunting (McGill and Sainsbury 2007).

The risk management options need to be assessed for feasibility and affordability so that they can be accepted or rejected by decision makers. Including risk management actions into a wildlife translocation risk assessment begins to merge the role of risk analyst and decision maker, which is not encouraged in many fields such as environmental protection and veterinary policy making. However, in reality, wildlife translocation teams are small and integrated. The team manages all aspects of the project and therefore have the expertise to be able to make sound judgements on risk management. The inclusion of risk management options and their impact on the risks considerably expands the relevance and usefulness of the risk analysis as a practical tool.

In the DRAHS project, the veterinarians and scientists who complete the disease risk analysis also undertake veterinary care and disease surveillance activities on the translocation project. The understanding of the identified risks, practical and realistic risk management interventions and disease surveillance data collected allows for the implementation of comprehensive risk management procedures. For example, very detailed risk management procedures have been implemented for the corncrake reintroduction project, which has proceeded since 2001, in response to the changing risk of coccidial disease year on year determined through disease surveillance (Sainsbury and Jaffe 2015).

## Risk Analysis as a Tool for Decision Making

Risk analysis does not give a single correct answer to a problem but is a step-by-step exercise using facts and data plus opinions and judgements from a broad variety of perspectives (Wooldridge 2000).

One of the most difficult problems faced by decision makers is that of deciding what constitutes an acceptable risk. In some situations, it may be relatively easy to show the benefits as wells as the risks associated with the course of action. In other situations, it may be difficult to attain agreement on what constitutes an acceptable risk even in situations where risk can be quantified objectively. Knowledge of costs and benefits is seldom shared equally between all stakeholders who will therefore have different perspectives of acceptability (Wooldridge 2000).

Zero risk is seldom, if ever, attainable, and some degree of risk is unavoidable. For this reason, deciding whether or not a particular risk is acceptable is generally a societal or political decision because the benefits of a particular activity for one stakeholder group may have adverse consequences for another (MacDiarmid and Pharo 2003; Thrusfield 2007). In a pool frog translocation, the risk assessors decided that more data on the presence or absence of potentially alien parasites in the source population should be sought through screening, before translocation proceeded. Only 33 adult or juvenile pool frogs had been tested for alien parasites from the source population and extinctions of amphibians due to disease associated with alien parasites had been reported (McGill et al. 2005). However, the pool frog reintroduction steering committee, having considered the costs and benefits, decided to proceed with translocation.

Disease risk analysis is only part of the decision making process when considering whether a wildlife translocation should proceed. Financial costs, public support, political approval and stakeholder endorsement will all be other contributors. It may be necessary to make difficult trade-offs between the biologically or epidemiologically optimal decision and these other drivers. This is one of the many values of risk analysis as these decisions can be transparently and comprehensively assessed in an evidence-based, scientifically accepted process.

In the DRAHS project, the results of the disease risk analysis have led to the suspension of two reintroductions and the relocation of the rearing facility for a third. The reintroduction of barberry carpet moths was discouraged because the moths had been in contact with exotic lepidoptera (Sainsbury 2007) and no reintroduction using that captive colony has taken place. The barrier between captive adders and exotic vipers was found to be inadequate and the DRA suggested that the captive adders should not be released and another approach to conservation should be taken (Beckmann et al. 2014a). None of the captive adders were released. In the cirl bunting project and greatest risk from disease was attributed to housing the cirl buntings in a zoological collection and a recommendation made to move the captive reared birds to a facility distant from the zoo (McGill and Sainsbury 2007). A new cirl bunting rearing facility was created close to the reintroduction site (Fountain et al. 2016). In other cases, the DRA or DRM has fundamentally influenced the management of the project, for example, the elimination of suspected alien parasites in the dormouse project already mentioned, the risk management actions undertaken for corncrakes and the screening of cranes for inclusion body disease virus prior to the reintroduction commencing (Sainsbury and Vaughan-Higgins 2012). To date, no major zoonotic or agricultural significant diseases have been identified in DRAHS projects as high risk of disease and greater repercussions might be expected with such infectious agents. High-risk hazards identified have all impacted on the project focus species and closely related sympatric species.

## Conclusions

Wildlife disease risk analysis processes are still very much developing, and there are still challenges to address. Some do not consider risk analysis as a valid scientific methodology. One editorial referred to the discipline as a ‘fad’ related more closely to developing a bureaucratic excuse that few outsiders can fathom than to intelligent decision making (Anderson 1994).

Indeed, very few animal health risk analyses have been published in peer-reviewed literature (MacDiarmid 2000), and even fewer wildlife disease risk analyses have been published, although some examples do exist (Hartley 2009; Hartley et al. 2012).

This lack of recognition and difficulty in publication means that relatively few scientists are working in the field of wildlife disease risk analysis creating a shortage of expertise. These factors conversely affect the level of funding available to support experts to undertake wildlife disease risk analysis.

Major steps forward have been achieved through the recent publication of guidelines for wildlife disease risk analysis (Jakob-Hoff et al. 2014). This publication confirms general recognition of the usefulness of this tool especially in a field where data are severely lacking and difficult and expensive to generate. Although these guidelines provide an important review and a description of the approaches and tools available to risk analysts, they do not provide a single standardised approach as the field of wildlife disease is so diverse and the problems being assessed so varied.

It may be more feasible to develop a standardised and structured approach to disease risk analysis for conservation translocations as the scope and purpose of the risk analysis is better defined. Risk analysts are also likely to also be risk managers and so the priorities and drivers of risk management are less complex. Some authors have described their approach to translocation risk analysis (Corn et al. 2001; Davidson et al. 1992; Neimanis et al. 2004; Sainsbury et al. 2012; Sainsbury et al. In Press). This paper reviews the important features of risk analysis and discusses how refinements of the process, made through practical application in the wildlife field, could be implemented specifically for conservation translocation disease risk analysis. As recognition and acceptance of the role of risk analysis as an important tool in evidence-based decision making develops, the limitations of finance and available expertise must be overcome so that disease risk analysis is required as a fundamental component of planning, authorisation and implementation of conservation translocation so that disease risks are thoroughly investigated, assessed and managed so the risk of repeating the mistakes of the past is reduced.
